# Model selection based on logistic regression in a highly correlated candidate gene region

**DOI:** 10.1186/1753-6561-1-s1-s114

**Published:** 2007-12-18

**Authors:** Hae-Won Uh, Bart JA Mertens, Henk Jan van der Wijk, Hein Putter, Hans C van Houwelingen, Jeanine J Houwing-Duistermaat

**Affiliations:** 1 Department of Medical Statistics and Bioinformatics, Leiden University Medical Center, Leiden, The Netherlands

## Abstract

Our aim is to develop methods for identifying a (causal) variant or variants from a dense panel of single-nucleotide polymorphisms (SNPs) that are genotyped on the evidence of previous studies. Because a large number of SNPs are in close proximity to each other, the magnitude of linkage disequilibrium (LD) plays an important role. Namely, highly correlated SNPs may hamper standard methods such as multivariate logistic regression due to multicolinearity between the covariates. Sequences of models with high dimension naturally raise questions about model selection strategies. We investigate three variable selection methods based on logistic regression. The penalties on stepwise selection were imposed using the Akaike's Information Criterion (AIC), and using the lasso penalty. Finally, a Bayesian variable-selection logistic regression model was implemented. The methods are illustrated using the simulated dense SNPs including the causal DR/C locus on chromosome 6. We also evaluate model selection in terms of average prediction error across nine replicates. We conclude that for the Genetic Analysis Workshop 15 (GAW15) data, the newly developed Bayesian selection method performs well.

## Background

When a large number of potentially causative sites have been determined, the next question is how to distinguish the sites that have a causal role from the ones that show disease association because of linkage disequilibrium (LD). Stepwise logistic regression has been suggested to identify the relative importance of variants at different sites [1]. In order to deal with high correlation between the single-nucleotide polymorphisms (SNPs), the lasso penalty was applied for model selection, which shrinks some coefficients to zero for sufficiently large penalty [2]. Subsequently, we contrast results with an explicit variable selection implemented within fully Bayesian framework.

## Methods

### Model selection using penalties

Let *y*_*i*_, *i *= 1,..., *m*, be the binary response variable and let *x*_*ij*_, *j *= 1,..., *p*, be the predictor variables. Further define the monotone logit transformation *η*_*i *_= *p*_*i*_/(1 - *p*_*i*_), where *p*_*i *_is the probability of observing *y*_*i *_= 1. For the logistic regression model $\eta_{i}=\beta_{0}+\sum_{j=1}^{p}\beta_{j}x_{ij}$, where *β*_0 _denotes the offset, the binomial log-likelihood *l *is

$$l(\beta )=\sum_{i=1}^{m}\left\{y_{i}\log p(x_{i};\beta )+(1-y_{i})\log(1-p(x_{i};\beta ))\right\}.$$

Here *β *= {*β*_0_, *β*_*j*_} and the vector *x*_*i *_includes the constant term 1.

In case of a large number of predictors, it is often desirable to determine a smaller subset with the strongest effects. Our first strategy is to consider stepwise selection with Akaike's Information Criterion (AIC) [2], as defined by AIC = -2*l*(*β*) + 2*k*. Here, *k *is the number of parameters included in the model, and AIC penalizes for the addition of parameters.

As an alternative we impose the so-called *lasso *penalty [2-4]. The above log-likelihood [Eq. (1.1)] can be modified as follows:

$$l^{\lambda_{s}}(\beta )=l(\beta )-\lambda_{s}{\left\|\beta \right\|}_{s}^{},$$

where *s *= 1 and ||*β*||_1 _= ∑_*j*_|*β*_*j*_|, the *L*_1 _norm of the parameter vector *β*. This lasso-type penalization can be useful for variable selection, because it shrinks some coefficients to zero. Note that only the *β*_*j *_values are subject to penalization, not the offset *β*_0_. An optimal *λ *can be determined by AIC and 10-fold cross-validation (CV).

From a Bayesian point of view, Eq. (1.2) can be seen as the posterior mode for combining a flat prior *β*_0_, and independently normally distributed *β*_*j *_values. Therefore, lasso parameter *λ*_1 _can be seen as the inverse of the variance of the prior. From this perspective, it becomes clear why Markov Chain Monte Carlo (MCMC) techniques can be applied for better handling of model uncertainty.

### Bayesian model selection

We implemented a fully Bayesian approach to variable selection for the logistic regression model, with hierarchical specification on the regression parameter vector and logit link on the class probabilities. A flat prior was assumed for the intercept term *β*_0_, and the *β*_*j *_values were independently modeled as $N(0,\frac{1}{\zeta }\nu^{2}I_{k})$-distributed random variables, where *ν *is a known scale and 1/*ζ *a rescaling factor, such that *ζ *has a Gamma(*a*, *b*) distribution with *a *and *b *positive real numbers. We assumed a uniform prior on SNP location choices (i.e., from a variable selection point of view, all SNPs have equal prior probability). An auxiliary variable approach was implemented to generate the logistic model via mixture modeling within an ordinary normal regression model [5,6]. A hybrid MCMC sampler was applied, based on random choices between three steps to add (birth), remove (death), or move a SNP to the model. For more details we refer to Mertens [7], Green [8], and Holmes and Held [6]. We performed a simulation of 100,000 iterations and discarded the first 50,000 as burn-in. The classification performance from the model was investigated based on the marginal mean posterior class probabilities. Sensitivities and specificities were presented along with the receiver operating characteristic (ROC) curve. The Bayesian logistic regression variable selection model was implemented in MATLAB.

### Evaluation of the model selection regarding prediction performance

Because it is difficult to defend a model that predicts poorly, we also examined prediction performance. The first simulated data set, Replicate 1, was used as a training set to determine the optimal set of variables, and Replicates 2 to 10 were used for validation by calculating average prediction error. This was defined to be the average of the classification errors from the predicted values using selected SNPs for each of nine data sets. The computations were performed with the programming language R modifying various existing packages.

### Materials

We used ten replicates of simulated data, a dense map of 17,820 SNPs on chromosome 6, modeled after the rheumatoid arthritis (RA) data. We selected a high LD region of approximately 5 Mb including the trait loci (DR/C locus) with a large trait effect; we knew the "answers". Setting a threshold of *p*-value < 0.001 using the Cochran-Armitage trend test, we obtained 73 SNPs. Further, we chose 200 cases and 200 controls by the RA status.

## Results

**Figure 1:**
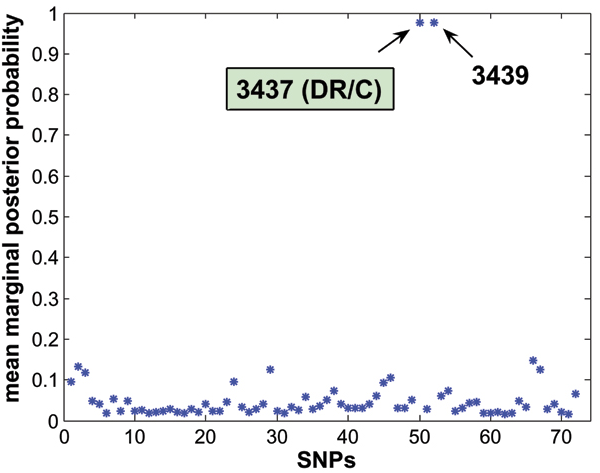
**Relative importance of the SNPs across all models simulated**. Two SNPs including the causal SNP 3437 were selected nearly 98% of all models.

**Figure 2:**
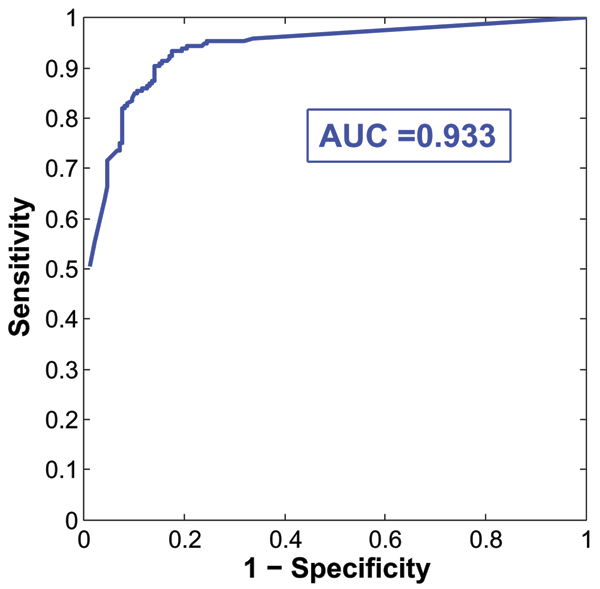
**Classification performance of all models simulated**. ROC curve for the marginal mean posterior class probability.

Whereas the disease SNP 3437 was selected by all methods (Table 1), the Bayesian logistic variable selection regression model was the most parsimonious one, as it identified only two SNPs. In Figure 1, the number of times that each particular SNP was selected into the model across all models simulated was expressed as a percentage of the total number of models considered. The two SNPs were selected from nearly 98% of all models. To summarize classification performance from all models visited, we calculated the mean posterior class probability, and found sensitivity and specificity of 0.82, with the global misspecification error of 0.13. Figure 2 shows the ROC curve for the marginal mean posterior class probability and the area under the curve (AUC) equals to 0.933.

**Table 1:** Selection of SNPs

Selection methods	No. selected SNPs	Selected SNPs
Stepwise/AIC	9	2823 3301 3379 3384 3394 **3437**^a ^3439 3474 3477
Lasso/AIC	29	2823 2826 2827 2848 2859 3286 3301 3310 3352 3366 3379 3384 3387 3394 3396 3426 3429 3430 **3437 **3439 3440 3447 3459 3474 3478 3481 3580 3581 3599
Lasso/CV	17	2823 2826 2827 2848 3301 3310 3379 3387 3394 3426 3429 3430 **3437 **3439 3440 3474 3599
Bayesian	2	**3437 **3439
Trait locus DR/C*		**3437**

^a ^The bold-typed 3437 is the trait locus DR/C.

Even though validation set size (nine replicates) was very small, we investigated prediction performance to evaluate the models (Table 2). Based on the average prediction error over nine replicates, the Bayesian selection seemed to outperform other methods.

**Table 2:** Evaluation of model selection

		Average Prediction Error (SE)^a^		
		
Selection methods	No. selected SNPs	Without regularization	Ridge penalty	Random forests
Stepwise/AIC	9	0.1536 (0.0049)	0.1506 (0.0044)	0.1586 (0.0068)
Lasso/AIC	29	0.1572 (0.0051)	0.1450 (0.0046)	0.1469 (0.0059)
Lasso/CV	17	0.1461 (0.0052)	0.1428 (0.0060)	0.1558 (0.0052)
Bayesian	2	**0.1306 (0.0052)**^b^	0.1306 (0.0052)	0.1336 (0.0043)
Trait locus DR/C*			0.1572 (0.0058)	

^a^Average prediction error was calculated from Replicates 2 to 10 using the optimally selected set of SNPs from Replicate 1.

^b^Bold indicates the minimum average prediction error.

## Discussion

When we dropped the causal SNP and analyzed data again, by stepwise selection with the AIC and by the lasso penalty method, the same remaining SNPs were selected. The corresponding average prediction error still remained at the same level. Meanwhile, the Bayesian method selected two SNPs, 3436 (with probability 87%) and 3439 (with probability 97%), between which the causal SNP 3437 was located. Because the penalty methods selected several (possibly correlated) SNPs, we applied ridge penalty [9] and random forests [10] to stabilize the system. We found that prediction performance generally improved slightly with ridge penalty regularization (Table 2). We also compared the above findings with situations in which only the causal SNP was included in the model. In all situations, prediction performance based on nine replicates remained almost at the same level for each selection method.

Additionally, we analyzed another candidate region of 5 Mb around the D locus with a small effect and moderate LD, where the causal SNP was not included. Using these data sets, which can be considered as the opposite of those used in the main analyses, none of the methods performed well in terms of prediction performance.

Our Bayesian method can (theoretically) deal with a great number of SNPs, provided that time and facilities are available. The computational efficiency is mainly achieved by integrating out regression coefficients within the ratio of marginal likelihoods. However, the usefulness for genome-wide scan remains to be evaluated.

## Conclusion

All methods identified the causal SNP together with other variants. In terms of parsimony of the model and prediction performance, Bayesian method outperformed other methods. When high correlations between the SNPs are characteristics in some candidate region such as in the specific data presented in this paper, and the focus of investigation is to find a causal gene, we conclude that a Bayesian method might perform well to disentangle the structure. Figures 1 and 2 summarize our evaluation results succinctly. The plot of mean posterior class probability indicates relative importance of the selected SNPs: i.e., in average how many times these SNPs were included across all the models simulated. Additionally, the ROC curve indicates how well these models were classified.

## Competing interests

The author(s) declare that they have no competing interests.
